# Biglycan and reduced glycolysis are associated with breast cancer cell dormancy in the brain

**DOI:** 10.3389/fonc.2023.1191980

**Published:** 2023-06-29

**Authors:** Ashley Sunderland, Jennifer Williams, Tereza Andreou, Nora Rippaus, Christopher Fife, Fiona James, Yolanda Dyah Kartika, Valerie Speirs, Ian Carr, Alastair Droop, Mihaela Lorger

**Affiliations:** ^1^ School of Medicine, University of Leeds, Leeds, United Kingdom; ^2^ School of Medicine, Medical Science and Nutrition, University of Aberdeen, Aberdeen, United Kingdom; ^3^ Experimental Cancer Genetics, Wellcome Sanger Institute, Hinxton, United Kingdom

**Keywords:** dormancy, breast cancer, brain metastases, glycolysis, biglycan, YAP

## Abstract

Exit of quiescent disseminated cancer cells from dormancy is thought to be responsible for metastatic relapse and a better understanding of dormancy could pave the way for novel therapeutic approaches. We used an *in vivo* model of triple negative breast cancer brain metastasis to identify differences in transcriptional profiles between dormant and proliferating cancer cells in the brain. *BGN* gene, encoding a small proteoglycan biglycan, was strongly upregulated in dormant cancer cells *in vivo*. *BGN* expression was significantly downregulated in patient brain metastases as compared to the matched primary breast tumors and *BGN* overexpression in cancer cells inhibited their growth *in vitro* and *in vivo*. Dormant cancer cells were further characterized by a reduced expression of glycolysis genes *in vivo*, and inhibition of glycolysis *in vitro* resulted in a reversible growth arrest reminiscent of dormancy. Our study identified mechanisms that could be targeted to induce/maintain cancer dormancy and thereby prevent metastatic relapse.

## Introduction

The vast majority of breast cancer deaths are due to metastases, which often develop years after the initial diagnosis of the primary tumor. Evidence suggests that non-proliferating, asymptomatic cancer cells that lay dormant in different organs since their initial dissemination may be responsible for metastatic relapse. Brain metastases develop in ~20% of cancer patients and are associated with a very poor prognosis (1, 2). Brain metastases tend to develop late in the course of progressive metastatic disease, and thus tend to have a longer latency period as compared to metastases at other sites (3). Therefore, cancer cell dormancy may be of a particular interest in the context of brain metastases. While several molecular players and pathways involved in the regulation of a dormant phenotype have been identified (4–6), our understanding of dormancy is still very limited, particularly when it comes to cancer dormancy in the brain (7). Better understanding of mechanisms involved in the regulation of dormancy may reveal unique opportunities for therapeutic interventions, for example by inducing dormancy in proliferating cancer cells.

We here used an *in vivo* model of experimental breast cancer brain metastasis to identify the molecular profile of dormant cancer cells in the brain. Our study reveals a functional relationship between reduced aerobic glycolysis and dormant phenotype, and an involvement of a small proteoglycan biglycan.

## Methods

### Cell culture

MDA-MB-231 and HEK293 cells were obtained from ATCC. MDA-MB-231 cells were cultured in EMEM (Sigma Aldrich) containing 10% FBS, L-glutamine, vitamin mix, non-essential amino acids, sodium pyruvate and penicillin/streptomycin. HEK293 cells were grown in DMEM (Sigma Aldrich) containing 10% FBS and penicillin/streptomycin. Cells were regularly tested for Mycoplasma and confirmed to be Mycoplasma free. Whenever specified, MDA-MB-231 cells were labeled using CellVue Claret^®^ Far Red Fluorescent dye (Sigma Aldrich) according to manufacturer’s instructions.

For *in vitro* assays, MDA-MB-231 cells were seeded at 1x10^5^ cells per 6-well. High glucose DMEM/F-12 medium (Thermofisher, 11320033) was used for experiments with 2-DG (Sigma Aldrich) and medium replenished daily.

### Generation of MDA-MB-231/BGN and MDA-MB-231/CON cells

Neomycin resistance gene was inserted downstream of PGK promoter in pTREAutoR3 lentiviral vector (8). *GFP* gene downstream of a doxycycline-inducible minimal CMV promotor was replaced with *BGN* gene. Vector without an insert was used as s control. Lentivirus was generated as previously described (9). Following transduction, MDA-MB-231 cells were maintained in medium containing TET Systems approved FBS (Thermofisher). Neomycin (500µg/ml) was added to the medium until all non-transduced cells perished. Cells transduced with pTREAutoR3_Neo_BGN and control vector were named MDA-MB-231/BGN and MDA-MB-231/CON, respectively. *BGN* expression was induced by adding doxycycline (1µg/mL) for at least 2 days. For *in vivo* experiments, cells were tagged with Firefly luciferase as described (9).

### *In vivo* studies

Six- to eight-week-old C.B.17 SCID mice (C.B-*lgh-1^b^/lcr*Tac-*Prkdc^scid^)* were purchased from Charles Rivers Laboratories. Brain tumor xenografts were generated by injection of 1x10^5^ CV-labelled MDA-MB-231 cancer cells into the internal carotid artery (10) or through implantation of firefly luciferase-tagged MDA-MB-231/BGN and MDA-MB-231/CON cancer cells (1x10^5^) into the striatum as previously described (9). Doxycycline (100 mg/kg BW) was administered i.p. on days 1, 3 and 5 post-cancer cell injection, and in water (2 mg/mL) from day 6 on. Tumor growth was monitored *in vivo* by bioluminescence imaging, using IVIS Spectrum (Perkin Elmer). Living Image software (Perkin Elmer) was used for quantification of bioluminescence signals.

### Ethical approval statement

All procedures were approved by the University of Leeds Animal Welfare & Ethical Review Committee and performed under the approved UK Home Office project license.

### Sorting of cancer cells from brains

Left hemisphere of the brains isolated from mice at 4 weeks post-intracarotid injection of CV-labeled GFP+ cancer cells were enzymatically dissociated in EMEM containing 3mg/ml collagenase and 250 U/ml hyaluronidase for 20 minutes at 37°C. Tissue was washed in cold incubation buffer (0.5% BSA and 2mM EDTA in PBS) and strained. Myelin was removed using Myelin Removal Beads II (Miltenyi Biotec). GFP+CV- and GFP+CV+ cancer cells were sorted from the resulting cell suspension using the Influx v7 Sorter (BD Biosciences). Cultured MDA-MB-231 cells (GFP-tagged, untagged CV-labelled, GFP-tagged and CV-labeled, and untagged/unlabeled) were used for compensation. Analysis was performed using FlowJo.

### RNA sequencing, data processing and analysis

Total RNA from sorted cancer cells was isolated using the Arcturus^®^ PicoPure™ RNA Isolation Kit (Thermo Fisher), followed by DNA removal using the RNase-free DNase Set (Qiagen). Smart-seq2 protocol (11) and the Nextera XT DNA Library Prep kit (Illumina) were used to generate full-length cDNA and sequencing libraries. PCR amplification was performed for 15 cycles. Libraries were pooled and paired-end mRNA sequencing was performed using the Hiseq3000 platform (Illumina).

Data processing was performed using R/Bioconductor. Reads were quality-assessed and trimmed using FastQC (12) and Cutadapt (13), respectively. Primary assemblies and comprehensive gene annotations for human, release 31 (GRCh38.p12), and mouse, release M22 (GRCm38.p6), were sourced from GENCODE (14). Human and mouse chromosomal identifiers were renamed for disambiguation, and assemblies concatenated using Biostrings (15). Reads were aligned using STAR aligner (16), with Qualimap (17) and Picard Tools (18) used for quality assessment. Read quantification, transcriptome merging and count mapping was performed using RSubread (19). Multi-mapping reads were included. Mouse-mapping reads, as identified from prior chromosomal renaming, were discarded as the desired cancer cells were of human origin. Read counts were converted to integers, and size factor normalization was performed using DESeq2 (20). Ensembl IDs were converted to gene symbols using Ensembl Gene ID Converter (21).

Genes were analyzed using an *FDR =< 0.05* cutoff, unless otherwise stated. Functionally implicated transcription factors were predicted using TFactS (22). Functional protein annotation networks were visualized using STRING (23). Heat maps (unsupervised hierarchical clustering), constructed at the transcript level, and principal component analysis (PCA) plots, were generated using ClustVis (24). Kyoto Encyclopedia of Genes and Genomes (KEGG) enrichment analysis was carried out using ClusterProfiler (25), with no output statistical cut-off, however the input gene list was restricted to a minimum fold change of 2.

### Data availability statement

The mRNAseq datasets generated for this study can be found in the Gene Expression Omnibus database with the accession code GSE220017. Publicly available data sets analyzed in this study included GSE2034, GSE5327, GSE12276, GSE14017 and GSE43837.

### Analysis of publicly available datasets

Raw data from GSE2034, GSE5327, GSE12276, GSE14017 and GSE43837 was extracted and independently normalized using the R package Affy (26). Gene expression data from Varešlija et al. were downloaded from the GitHub repository rpriedig (27). Pre-normalized *BGN* gene expression data was further normalized to that of *POLR2A* expression prior to analysis.

### Taqman qPCR assay

Total RNA from cells grown *in vitro* was isolated using the RNeasy Mini Kit (Qiagen). cDNA synthesis was performed with Superscript III Reverse Transcriptase kit (Invitrogen). Taqman PCR was performed as previously described (9). All assays were from ThermoFisher: *BGN* (Hs00959143_m1), *GAPDH* (Hs02786624_g1), *ITGB1* (Hs01127536_m1), *ITGB2* (Hs00164957_m1), *ITGB4* (Hs00236216_m1), and *POLR2A* (HS00172187_m1).

### Western blotting

Cell lysis and Western blot were performed as previously described (9). Primary antibodies were directed against biglycan (Proteintech, 16409-1-AP; 1:800), YAP1 (Santa Cruz Biotechnology, sc-101199; 1:1000), phospho-YAP1 (Fisher scientific, PA5-17481; 1:1000), and vimentin (DAKO, M0725; 1:1000). HRP-conjugated secondary anti-mouse and anti-rabbit antibodies were from Cell Signalling. Band intensity was quantified using Fiji image processing package.

### Cell cycle analysis and flow cytometry

Cells were incubated for 30 minutes with 10μM BrdU prior to harvest, and re-suspended in PBS, followed by dropwise addition of 9x volume of cold 70% ethanol and incubation on ice for 30 minutes. Cells were first re-suspended in denaturation buffer (PBS, 2M HCl, 0.5% Triton X-100) for 30 minutes at RT, followed by incubation in neutralization buffer (PBS, 0.1M Na_2_B_4_O_7_.10H_2_O, pH 8.5) for 30 minutes at RT, and then re-suspended in PBS, 1% w/v BSA, 0.5% v/v Tween-20 containing anti-BrdU-APC antibody (eBioscience, 17-025-152), and incubated for 1 hour at RT. Cells were washed 3x in PBS and incubated in PBS with 5μg/ml RNase A (Qiagen) and 10μg/ml propidium iodide (Sigma Aldrich) for 30 minutes at RY prior to flow cytometry analysis using the CytoFLEX Flow Cytometer (Beckman Coulter).

### Immunofluorescence

Cells were grown on plastic and fixed in 4% PFA for 10 minutes at RT. Mouse brain tissue was fixed and processed for floating sections, and staining performed as previously described (9). Incubation with all primary and secondary antibodies was for 1 hour at RT. Primary antibodies were directed against biglycan (Proteintech, 16409-1-AP; 1:200), GFP (Abcam, Ab13970; 1:1000), and YAP1 (Novus Biologicals, NB110-58358; 1:50). Secondary antibodies were from Jackson Immunoresearch. Nuclei were stained with DAPI. Images were acquired using AxioCam MRm (Zeiss) and AxioVision software (Zeiss), or the A1R confocal microscope equipped with Confocal NIS-Elements software (Nikon).

### Statistical analysis

Statistical analyses of data not derived from mRNAseq outputs were carried out using GraphPad Prism version 8.0.0 for Windows (GraphPad). The error bars on all graphs represent the standard error of the mean (SEM). Statistical significance between the experimental groups was determined by t-test or One-way ANOVA followed by multiple comparison analysis, as specified in figure legends.

## Results

### Dormant and proliferating cancer cells isolated from the brain have distinct molecular profiles

**Figure 1:**
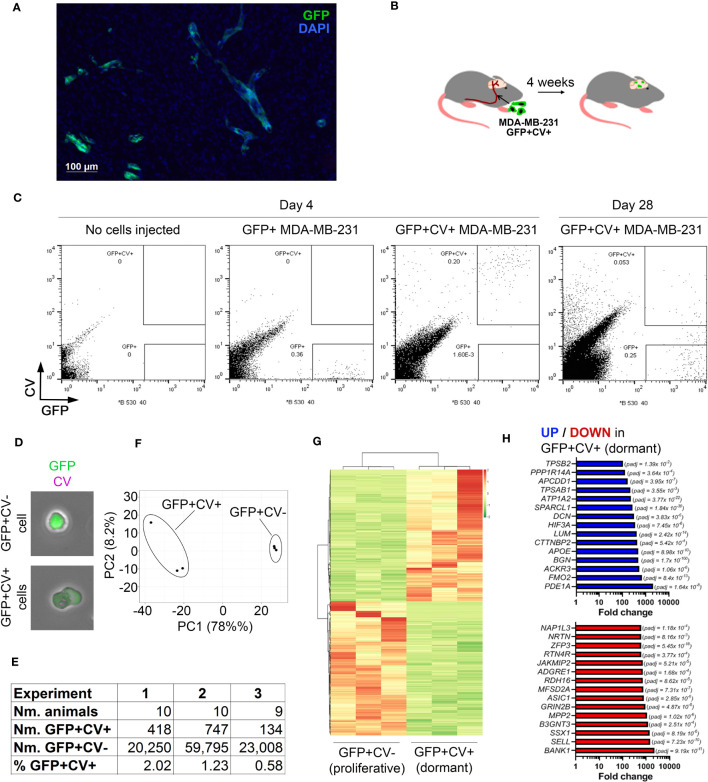
*In vivo* model of breast cancer cell dormancy in the brain and the molecular profile of dormant cancer cells. **(A)** Detection of cancer lesions in mouse brain 4 weeks after intracarotid injection of green fluorescence protein (GFP)-tagged MDA-MB-231 breast cancer cells by immunofluorescence. **(B)** Experimental scheme of the *in vivo* model for studying dormant and proliferating cancer cells in the brain. CellVue Claret (CV)-labelled, GFP-tagged MDA-MB-231 cancer cells were injected into the internal carotid artery and the brains isolated 4 weeks later. **(C)** Representative dot plots showing the analysis of mouse brains by flow cytometry. A control brain (no cells injected) and brains of mice that received GFP+ or CV-labelled GFP+ MDA-MB-231 cells, respectively, were harvested at 4 days post-cancer cell injection. A dot plot showing the analysis of pooled brains (*N=10*) isolated from mice receiving CV-labelled GFP+ MDA-MB-231 cells at 28 days post-cancer cell injection is displayed to the right. 54,000 events per plot are displayed. **(D)** GFP+CV- and GFP+CV+ cancer cells isolated from mice brains by FACS were analyzed by immunofluorescence. **(E)** Experimental details of 3 independent *in vivo* experiments for isolation of dormant and proliferating cancer cells. **(F)** Principal component analysis (PCA) of dormant and proliferating cancer cell samples from 3 independent *in vivo* experiments based on differentially expressed genes. **(G)** Heat map visualizing hierarchical clustering of genes differentially expressed between dormant and proliferating cancer cells.**(H)** Top 15 up-regulated (top panel; blue) and down-regulated (bottom panel; red) genes in dormant (GFP+CV+) versus proliferating (GFP+CV-) cancer cells.

We previously reported that, following its injection into the internal carotid artery, the triple negative breast cancer cell line MDA-MB-231 displayed a low efficiency of cancer cell outgrowth in the brain following initial cancer cell seeding (Lorger and Felding-Habermann, 2010). Analysis of coronal brain sections by immunofluorescence 4 weeks after the injection of green fluorescence protein (GFP)-tagged MDA-MB-231 cells confirmed the presence of cancer cells in the brain, either as small cell clusters or single cells, with very few larger cancer lesions observed (**Figure 1A**). To distinguish between cancer cells that have undergone proliferation following their arrival in the brain versus those that remained dormant, GFP+ cancer cells were labelled with CellVue Claret (CV) vital dye. We confirmed *in vitro* that in proliferating cultured MDA-MB-231 cells this dye is diluted below the limit detectable by flow cytometry within 14 days, as the dye is being equally split between daughter cells at each cell division (**Supplementary Figure 1A**). As such CV loss can be used to detect proliferating cells, while non-proliferating cells are expected to retain the dye.

CV-labelled GFP+ MDA-MB-231 cells were injected into the internal carotid artery of CB17SCID mice (**Figure 1B**). We have previously demonstrated that cancer cells start extravasating into the brain at ~day 3 post-injection and all cells are extravascular by day 7 (10). To determine whether GFP+CV+ cancer cells can be detected in the brain prior to resuming proliferation, we isolated and dissociated whole brains 4 days post-cancer cell injection and analyzed them by flow cytometry. At this early time point, all cancer cells appeared within the GFP+CV+ gate and with no events observed within the GFP+CV- gate. The latter was set based on the analysis of brains isolated from mice following the injection of GFP+ cancer cells not labelled with CV and based on cultured GFP+, CV+ and GFP+CV+ MDA-MB-231 cells (**Figure 1C**; **Supplementary Figure 1B**). This confirmed a reliable separation of GFP+CV- and GFP+CV+ cancer cell populations in mouse brains by flow cytometry.

We next isolated by FACS non-proliferating dormant (GFP+CV+) and proliferating (GFP+CV-) MDA-MB-231 cancer cells from the mice brains 4-weeks after the injection of GFP+ cancer cells labeled with CV into the internal carotid artery. Due to the low number of cancer cells in the brain, 9-10 mice brains were pooled. The majority of cancer cells lost CV dye and appeared within the GFP+CV- gate as expected, as proliferating cells outnumbered any dormant cells within the 4-week period due to their expansion. However, a clear population of GFP+CV+ cancer cells remained (**Figure 1C**, right). We also confirmed that the cells were GFP+CV- or GFP+CV+, respectively, by immunofluorescence (**Figure 1D**). To obtain biological triplicates, 3 independent experiments were performed, and isolated cells subjected to the genome-wide gene expression analysis by mRNAseq. As summarized in **Figure 1E**, GFP+CV+ cancer cells represented between 0.58 to 2.02% of all isolated GFP+ cancer cells.

Principal component analysis (PCA) revealed separate clustering of dormant and proliferating cell transcriptomes (**Figure 1F**). Unsupervised hierarchical clustering of samples identified 1161 genes that were differentially expressed between dormant and proliferating cancer cells (*FDR* < 0.05) (**Figure 1G**; **Supplementary Table IA**). The most significantly differentially expressed gene was *BGN* (*FDR* 1.7x10^-100^), which encodes a small extracellular matrix protein biglycan (28), with 474-fold upregulation in dormant versus proliferating cancer cells (**Figure 1H**).

To determine whether any of the previously identified dormancy markers (29) are associated with a dormant cancer cell phenotype in the brain, we analyzed the expression of these markers in our data set. While overall dormancy-associated markers were enriched in GFP+CV+ MDA-MB-231 cells isolated from the brains, this was not the case for all markers (**Supplementary Figure 1C**; **Supplementary Table IB**), suggesting a microenvironment and/or cancer type-dependent regulation of dormancy.

### Biglycan expression is associated with dormancy and inhibition of cancer cell growth *in vivo*

**Figure 2:**
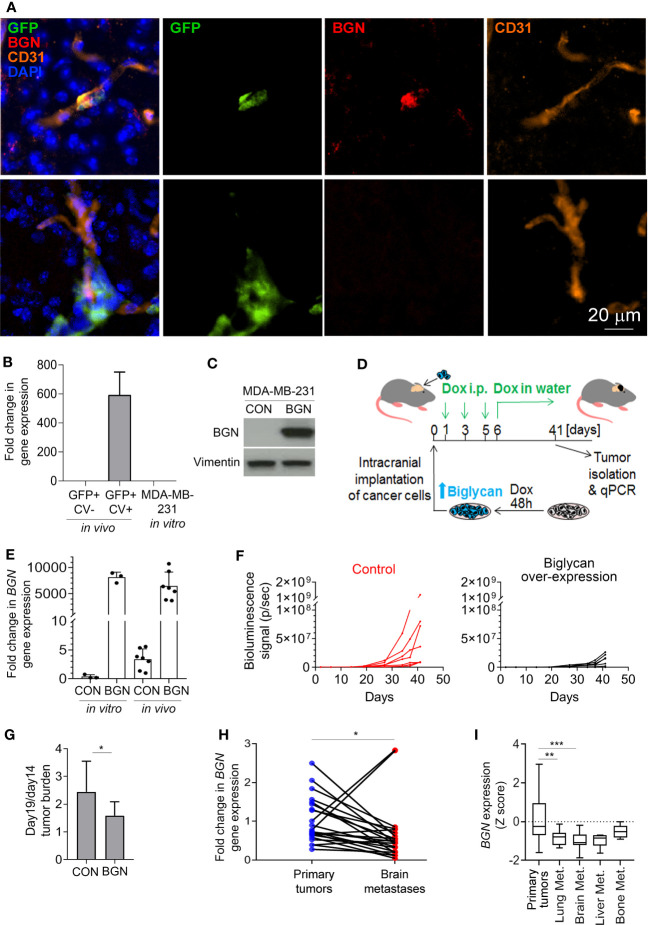
Biglycan inhibits cancer cell growth and is downregulated in proliferating brain metastases. **(A)** Immunofluorescence staining for biglycan (red), GFP+ cancer cells (green), and CD31+ blood vessels (orange) of mouse brain tissue isolated at 4 weeks post-intra-carotid injection of MDA-MB-231 cancer cells. Nuclear stain is shown in blue. Top row: single cancer cell (BGN+). Bottom row: a group of cancer cells (BGN-). **(B)** *BGN* mRNA expression (qPCR) *in vivo* in dormant and proliferating MDA-MB-231 cancer cells, and in MDA-MB-231 cell line grown *in vitro* in 10% serum. Error bars represent standard error. **(C)** Doxycycline-inducible *BGN* overexpression in MDA-MB-231/BGN cells exposed to 1 µg/mL doxycycline for 72 hours, detected by Western blot. No BGN can be detected in MDA-MB-231 control (CON) cells transduced with an empty vector. **(D)** Experimental scheme of an *in vivo* experiment. **(E)** Quantification of *BGN* gene expression (qPCR) in MDA-MB-231/CON and MDA-MB-231/BGN cells following Dox administration *in vitro* and in tumors of receiving Dox (*in vivo)*. **(F)** *In vivo* growth of intracranial tumors (bioluminescence signal) generated from MDA-MB-231/CON and MDA-MB-231/BGN cancer cells (*N=7* per group). **(G)** Increase in intracranial tumor burden following the initial tumor growth lag phase. Difference in bioluminescence signal between days 14 and 19 is show. Statistical significance was determined by one-tailed t-test with unequal variance (*≤ 0.05). **(H)** and **(I)** Analysis of *BGN* expression in publicly available gene expression data sets from Varešlija et al. **(H)** and from GSE2034, GSE5327, GSE12276, GSE14017 and GSE43837 **(I)** (27, 30–35). Statistical significance in H was determined by paired two-tailed t-test and in I by one-way ANOVA with Tukey’s multiple comparisons test (**≤ 0.01, ***≤ 0.001).

While most MDA-MB-231 cancer cells in the brain were biglycan-negative at 4 weeks post-cancer cell injection as analyzed by immunofluorescence, we detected rare biglycan-positive GFP+ cells (**Figure 2A**), confirming the existence of biglycan-expressing cancer cells *in vivo* at the protein level.

Similarly to proliferating cancer cells isolated from the murine brain, *BGN* was also absent from the fast proliferating MDA-MB-231 cells grown in 10% serum *in vitro* (**Figure 2B**). To investigate whether biglycan is functionally implicated in the regulation of cancer cell growth *in vivo*, we generated MDA-MB-231 cells stably expressing *BGN* (MDA-MB-231/BGN) under the control of a doxycycline-inducible promoter. We used an inducible promoter because *BGN* over-expression under a strong constitutive promoter resulted in cell growth arrest and precluded cell expansion (data not shown). Cells transduced with an empty vector were used as a control (MDA-MB-231/CON). Upon addition of doxycycline, a strong induction of biglycan could be detected in MDA-MB-231/BGN cells by Western blot in contrast to biglycan-negative MDA-MB-231/CON cells (**Figure 2C**). Firefly luciferase-tagged MDA-MB-231/CON and MDA-MB-231/BGN cells cultured in the presence of Dox for 2 days were subsequently implanted into the brain of CB17SCID mice (**Figure 2D**). Biglycan expression was maintained by doxycycline administration *in vivo* for the duration of the study. *BGN* expression in tumors was analyzed by qPCR at the endpoint and confirmed to be comparable to *in vitro* expression levels (**Figure 2E**). We observed a delayed tumor growth in mice with *BGN*-expressing tumors as compared to the control (**Figure 2F**; **Supplementary Figure 2**). This appeared to be due mainly to a delay in the cancer cell outgrowth following the initial 2-week lag-phase, as demonstrated by a significantly lower difference in the tumor burden increase between days 14 and 19 in the *BGN* over-expressing group (**Figure 2G**). This suggested that BGN may be primarily inhibiting the initial outgrowth of cancer cells. Notably, *BGN* overexpression also significantly inhibited the growth of MDA-MB-231 cells *in vitro* (**Supplementary Figure 1D**).

To establish a potential role of *BGN* in brain metastases in breast cancer patients, we analyzed publicly available gene expression data. This revealed a significant downregulation of *BGN* in brain metastases as compared to the patient-matched primary breast tumors (**Figure 2H**). Further analysis of non-matched primary breast tumors and breast cancer metastases from different organs revealed a significantly downregulated *BGN* expression in lung and brain metastases, and a tendency towards reduced *BGN* expression in liver metastases (**Figure 2I**). This demonstrated that cancer cells that can proliferate in the brain and develop into large metastases are associated with reduced *BGN* expression levels in patients, in line with our data in a preclinical model.

### Downregulation of glycolysis is associated with dormancy *in vivo* and an induction of reversible growth arrest *in vitro*

**Figure 3:**
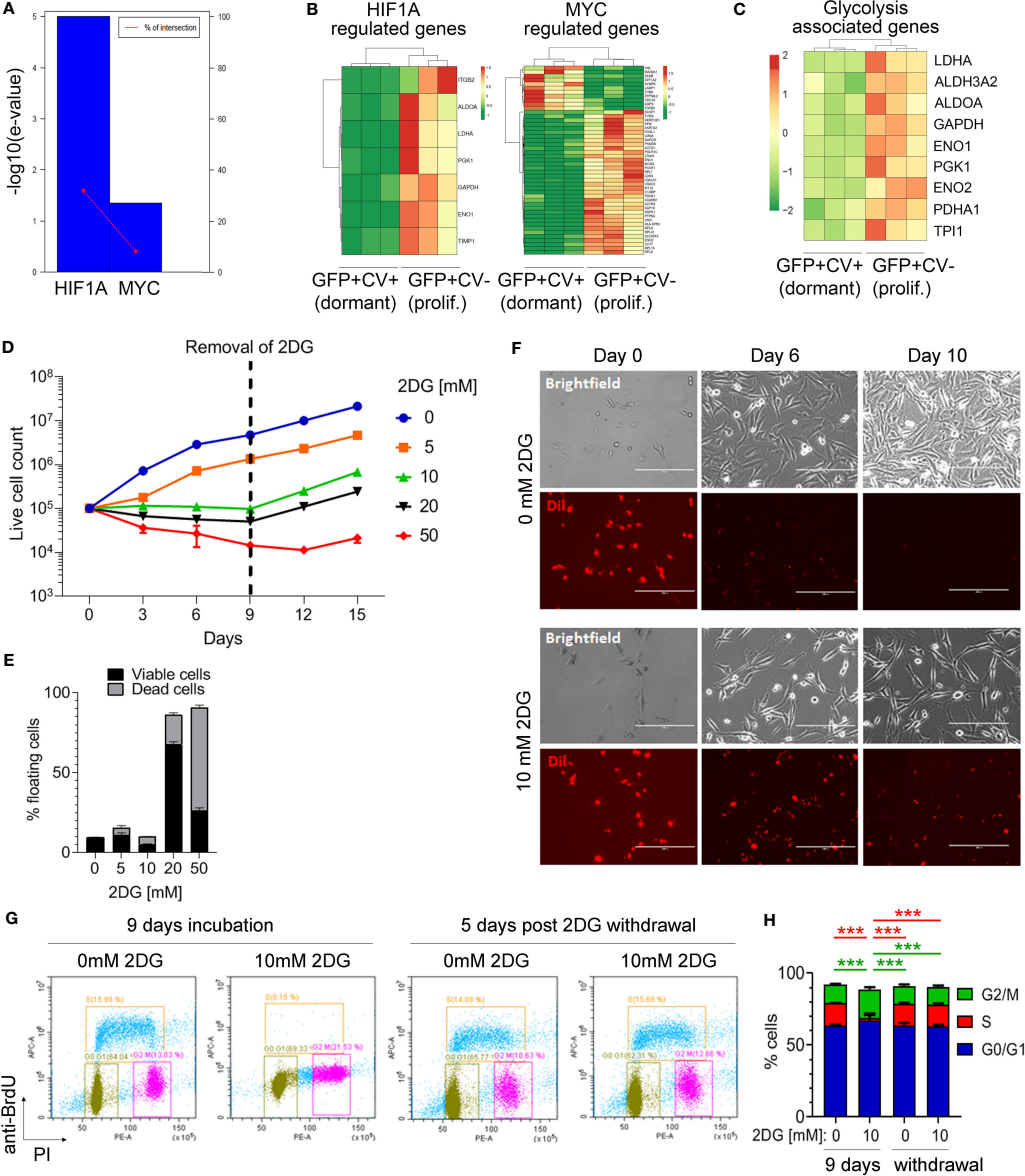
Inhibition of glycolysis is associated with cancer cell dormancy. **(A)** Genes differentially expressed between cell cycle-inactive and proliferating cancer cells *in vivo* were analyzed by TFacts software to identify statistically significantly repressed or activated transcription factors. Sign-sensitive analysis of differentially expressed genes (*FDR*<0.05) is shown. **(B)** Heat maps showing differential expression of HIF1α and MYC-regulated genes between the dormant and proliferating cancer cells. **(C)** Heat map showing hierarchical clustering of glycolysis-associated genes. **(D)** Growth curves of MDA-MB-231 cells cultured in 10% serum in the presence of different concentrations of 2-DG or vehicle (0 mM 2-DG). Dotted line marks the time point of 2-DG withdrawal. **(E)** Percentage of floating MDA-MB-231 cells following a 9-day incubation with different 2-DG concentrations. Proportion of viable and dead floating cells is shown in black and grey, respectively. **(F)** Immunofluorescence and light microscopy images of Dil-labelled MDA-MB-231 cells in a 10% serum-containing medium, visualizing loss of CV in the absence of 2-DG and CV retention in the presence of 10 mM 2-DG. **(G)** Analysis of the cell cycle by flow cytometry following a 9-day incubation with 10 mM 2-DG and a subsequent 2-DG withdrawal for 5 days. Control cells (0 mM 2-DG) have received vehicle only. Bromodeoxyuridine (BrdU) and propidium iodide (PI) were used to gate cells according to the cell cycle phase: G0/G1 (green gate), S (orange gate), G2/M (purple gate). **(H)** Quantification of cell cycle analysis shown in **(G)** Error bars represent standard error. One representative experiment out of three (each containing technical triplicates) is shown. Statistical differences were determined by one-way ANOVA followed by multiple comparison analysis (***≤ 0.001).

To identify potential differences in the activity of transcription factors between dormant and proliferating cancer cell populations, differentially expressed genes were analyzed using TFactS software (22). This revealed that HIF1α and MYC were significantly repressed in dormant as compared to the proliferating cancer cells (**Figure 3A**). Accordingly, HIF1α and MYC-dependent genes were differentially expressed between dormant and proliferating cancer cell samples (**Figure 3B**). Both transcription factors have been shown to induce a metabolic reprogramming towards aerobic glycolysis (36). Notably, nine of the HIF1α and MYC regulated genes that were downregulated in dormant cells are known to be involved in the regulation of glycolysis (**Figure 3C**). Thus, to further investigate whether inhibition of glycolysis induces reversible cell growth arrest in breast cancer cells, MDA-MB-231 cells were incubated with different concentrations of glucose analogue 2-deoxyglucose (2-DG). This reduced the growth rate of cancer cells in a dose-dependent manner (**Figure 3D**). Notably, at 10 mM 2-DG concentration, the number of live cells remained constant over a course of 9 days (**Figure 3D** **)** and only a low number of floating cells was observed, comparable to the control without 2-DG (**Figure 3E**). In contrast, higher 2-DG concentrations (20 and 50 mM) increased the proportion of dead floating cells, while lower 2-DG concentration (5 mM) failed to inhibit cell growth completely. Importantly, when 2-DG was removed to restore glycolysis, cancer cells resumed proliferation (**Figure 3D**), demonstrating that inhibition of glycolysis with low 2-DG concentration (10 mM) results in a reversible growth arrest in the absence of increased cell death, as seen in dormancy. In line with this, cancer cells retained CV dye when cultured in the presence of 10 mM 2-DG, while in the absence of 2-DG the CV was lost within 10 days (**Figure 3F**), mimicking our *in vivo* observations.

Inhibition of glycolysis with 10 mM 2-DG for 9 days significantly increased the percentage of MDA-MB-231 cells in G2/M phase as compared to control and reduced the percentage of cells in the S phase, while the proportion of cells in G0/G1 phase remained unaltered (**Figures 3G, H**). This cell cycle arrest was reversible following 2-DG removal, with percentages of cancer cells in different cell cycle phases returning to control levels at 5 days post-2-DG removal (**Figures 3G, H**). This suggested that inhibition of glycolysis is causing a reversible growth arrest of cancer cells in G2/M phase.

### Hippo signaling pathway is activated in dormant cancer cells *in vivo*

**Figure 4:**
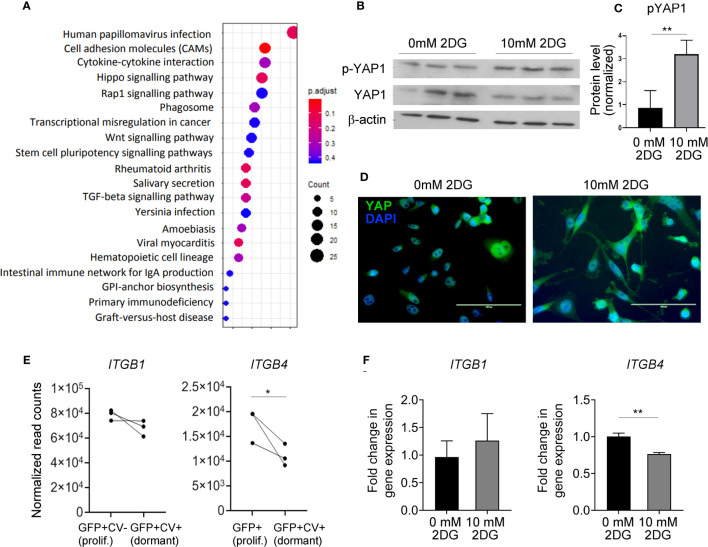
Hippo pathway is implicated in dormant phenotype. **(A)** KEGG enrichment analysis of genes differentially expressed between dormant and proliferating cancer cells. **(B)** Western blot analysis of YAP1 and phospho (p)-YAP1 expression levels in MDA-MB-231 cells exposed to 0 or 10 mM 2-DG for 48 hours. One representative experiment containing biological triplicates out of three independent experiments is shown. **(C)** Quantification of Western blot shown in **(B)** Signal for phosphorylated YAP was normalized to the signal for total YAP. **(D)** Cellular localization of YAP following the exposure of MDA-MB-231 cells to 0 or 10 mM 2-DG for 48 hours. **(E)** mRNA expression levels of the indicated integrin subunits in dormant and proliferating MDA-MB-231 cells isolated from the brain. **(F)** *ITGB1* and *4* gene expression in MDA-MB-231 cells exposed to 0 or 10 mM 2-DG for 48 hours. Statistical significance in C, E and F was determined by unpaired two-tailed t-test with unequal variance (*≤ 0.05, **≤ 0.01).

KEGG analysis on genes differentially expressed between dormant and proliferating cancer cells isolated from the mouse brains identified several differences (**Figure 4A**; **Supplementary Table II**). Hippo signaling pathway was amongst the top enriched pathways in dormant cells (**Figure 4A**), with Scribble planar cell polarity protein (*SCRIB*) and Disks large homolog 3 (*DLG3*), two upstream activators of Hippo pathway (37, 38), being significantly upregulated in dormant as compared to the proliferating cells (5.1- and 2.1-fold change, respectively; **Supplementary Table I**). Transcription factor Yes associated protein (YAP) is at the core of the Hippo signaling pathway. As glycolysis has been previously implicated in the regulation of YAP activity (39) and we have demonstrated that mild inhibition of glycolysis induces reversible cancer cell growth arrest reminiscent of dormancy, we sought to further investigate a functional link between glycolysis and Hippo pathway in this context. When Hippo pathway is activated, YAP becomes phosphorylated, which prevents its translocation into the nucleus and blocks YAP-dependent gene transcription and proliferation (37). Inhibition of glycolysis with 10 mM 2-DG resulted in a significant increase in YAP phosphorylation (**Figures 4B, C**) and its cytoplasmic retention (**Figure 4D**), suggesting that inhibition of glycolysis leads to Hippo activation. This further suggests that downregulated glycolysis may induce dormancy through Hippo pathway *in vivo*.

Notably, YAP is involved in the regulation of cell adhesion to the extracellular matrix by regulating the expression of various integrins. We observed a significant downregulation of *ITGB4* in dormant as compared to the proliferating cancer cells *in vivo*, and in 2-DG-treated as compared to vehicle-treated cancer cells *in vitro* (**Figures 4E, F**), while the expression of *ITGB1* was unaltered. This suggests that glycolysis may be involved in dormancy potentially by regulating integrin expression and consequently cell adhesion to the basement membrane.

## Discussion

Our study reveals novel mechanisms involved in the dormancy of breast cancer cells in the brain. *BGN* was the most significantly upregulated gene in dormant breast cancer cells in our model and was downregulated in actively growing brain and lung metastases as compared to the primary tumors in patients. Our *in vivo* data suggests that biglycan may be inhibiting the initial outgrowth of cancer cells in the brain. In line with our findings, biglycan has been previously shown to induce breast cancer cell normalization, as indicated by the induction of acinar spheroid formation and reduced proliferation (28), to induce cell cycle arrest in pancreatic cancer cell lines (40), and to inhibit growth of bladder cancer cells (41).

Hippo pathway was one of the most significantly upregulated pathways in dormant cancer cells in our model. Activation of YAP has been previously demonstrated to promote metastasis (42). YAP activation has been also involved in the outgrowth of disseminated cancer cells in multiple organs, with β1 integrin-mediated signaling playing a key role (43–45). During the outgrowth of disseminated cancer cells in different organs, including the brain, the YAP activation was induced by L1CAM-dependent cancer cell spreading on the vasculature, through activation of β1 integrin and ILK (44). In our model, *ITGB4* gene (encoding β4) was significantly downregulated in dormant cancer cells *in vivo*, while *ITGB1* (encoding β1) expression remained unaltered. We did however not investigate the integrin activation state and thus it is possible that β1 integrin also plays a role in our dormancy model. While it would have been interesting to determine whether dormant cancer cells in our model display deficiency in spreading, the low frequency of dormant events *in vivo* precluded such analysis. Our study focused instead on a stimulus different to cell spreading, namely glycolysis. Our data revealed that reduced glycolysis inhibits YAP and induces reversible cancer cell growth arrest reminiscent of dormancy. Inhibition of glycolysis significantly enhanced YAP phosphorylation and its cytoplasmic retention, suggesting that glycolysis-dependent YAP regulation occurs via the canonical Hippo pathway, although this would require further experimental confirmation. In contrast to this, Er et al., reported that L1CAM knockdown inhibited YAP transcriptional activity without affecting YAP phosphorylation or upstream Hippo pathway kinases (44). Moreover, a recent study demonstrated that dystroglycan receptor sequesters YAP from the nucleus in quiescent disseminated cancer cells in the brain (46). In summary, this implies that multiple positive and negative signals converging on YAP via different upstream pathways may drive dormant/latent versus proliferative cancer cell state.

Our study provides an insight into some of the molecular players involved in the regulation of cancer cell dormancy in the brain, and raises additional important questions requiring further investigations, such as the identity of stimuli that repress HIF1α and MYC activity, and the role of other pathways significantly enriched in dormant cells that were not investigated in this brief report. Based on our study, therapeutic interventions leading to the maintenance of cancer cell growth arrest can be envisioned, such as inhibition of glycolysis by 2-DG and its analogues, which are currently being considered as anti-cancer drugs (47), or YAP inhibitors, from which several are already in clinical trials (48). As our study shows that multiple molecular players contribute to cancer cell growth arrest in the brain, it is likely that strategies targeting multiple pathways will be required for the maintenance of dormancy in brain metastases.

## Data availability statement

The datasets presented in this study can be found in online repositories. The names of the repository/repositories and accession number(s) can be found in the article/**Supplementary Material**.

## Ethics statement

The animal study was reviewed and approved by Animal Welfare and Ethics Review Committee, University of Leeds.

## Author contributions

Conceptualization, ML. Methodology, ML, AS, IC, AD. Investigation, AS, JW, TA, NR, CF, FJ, YD. Writing – Original Draft, ML, AS. Writing – Review & Editing, ML, AS, JW, TA, CF, FJ, VS. Supervision, ML, IC, AD, VS. Funding Acquisition, ML. All authors contributed to the article and approved the submitted version.

## Supplementary material

Supplementary Figure 1
**(A)** Loss of CellVue Claret (CV) dye through proliferation was confirmed by flow cytometry through analysis of GFP+ CV-labelled MDA-MB-231 cells cultured *in vitro* (top panel). GFP+ cells without CV label were used as a control (bottom panel). **(B)** Flow cytometry plots showing cultured MDA-MB-231 cells (untagged, GFP+, CV+, GFP/CV double positive) with the gate setting used for sorting of MDA-MB-231 cells from mouse brains. **(C)** Expression of genes previously identified as dormancy markers in various contexts was analyzed in dormant and proliferating cancer cells isolated from the brains in our MDA-MB-231 model. Unsupervised hierarchical clustering of samples demonstrates a clear separation of dormant and proliferative cancer cell populations. **(D)** Quantification of *in vitro* growth, comparing MDA-MB-231/BGN and MDA-MB-231/CON cells.

Supplementary Figure 2 Bioluminescence images used to quantify intracranial growth of tumors generated from MDA-MB-231/CON and MDA-MB-231/BGN cancer cells, showing different days post-cancer cell implantation as indicated. Due to a strong increase in signal intensity over time, different signal intensity scales were used for displaying images taken on days 6-22, days 27-34, and days 37-41, respectively, to allow for visualization of signals at all time points.
