# Supplementary figures and images for: Biglycan and reduced glycolysis are associated with breast cancer cell dormancy in the brain
Source: Front Oncol. 2023 Jun 29;13:1191980. doi: 10.3389/fonc.2023.1191980 (PMC10339804; doi:10.3389/fonc.2023.1191980)

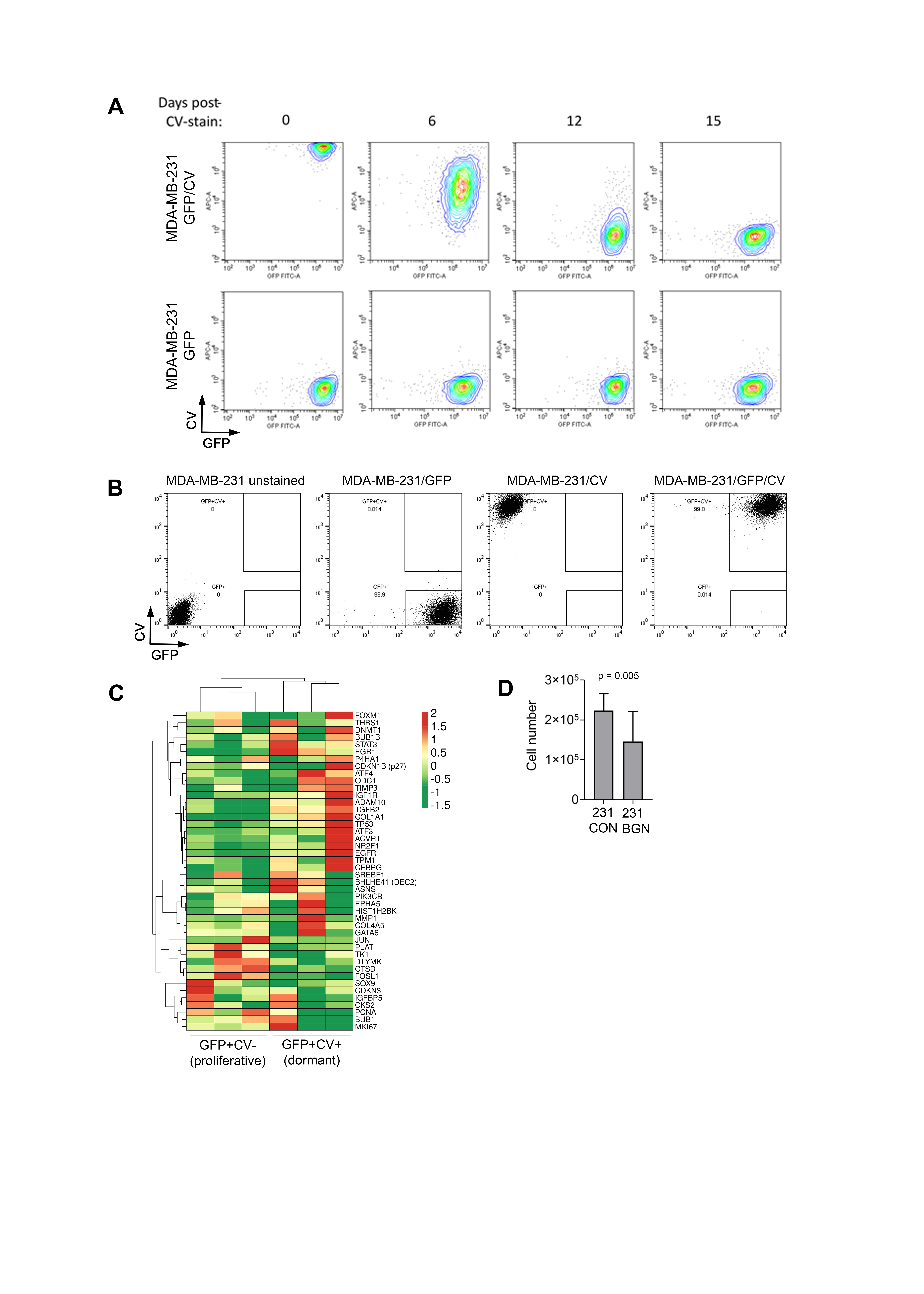

Supplement: Supplementary Figure 1 — (A) Loss of CellVue Claret (CV) dye through proliferation was confirmed by flow cytometry through analysis of GFP+ CV-labelled MDA-MB-231 cells cultured in vitro (top panel). GFP+ cells without CV label were used as a control (bottom panel). (B) Flow cytometry plots showing cultured MDA-MB-231 cells (untagged, GFP+, CV+, GFP/CV double positive) with the gate setting used for sorting of MDA-MB-231 cells from mouse brains. (C) Expression of genes previously identified as dormancy markers in various contexts was analyzed in dormant and proliferating cancer cells isolated from the brains in our MDA-MB-231 model. Unsupervised hierarchical clustering of samples demonstrates a clear separation of dormant and proliferative cancer cell populations. (D) Quantification of in vitro growth, comparing MDA-MB-231/BGN and MDA-MB-231/CON cells. [file Image_1.jpeg]

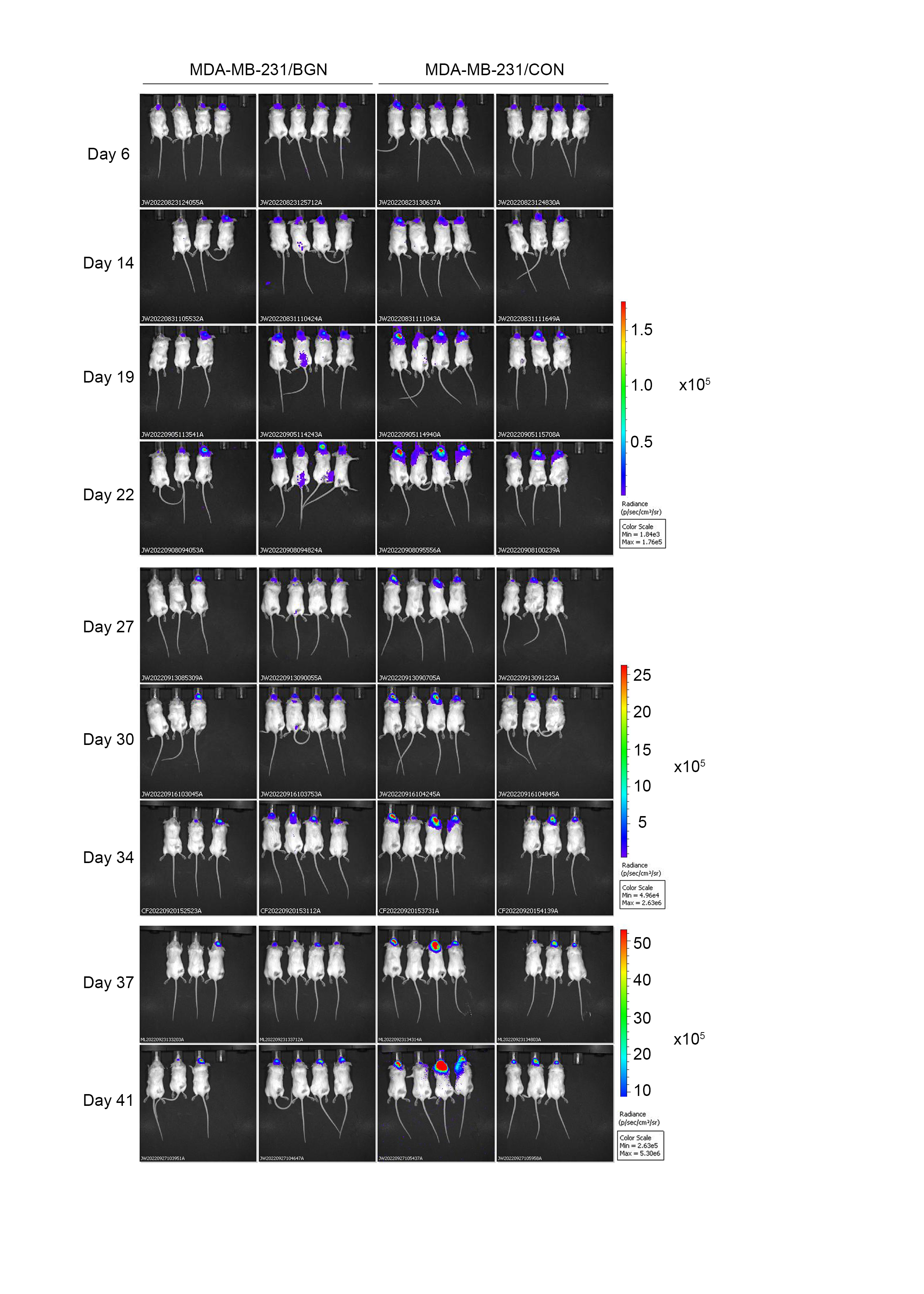

Supplement: Supplementary Figure 2 — Bioluminescence images used to quantify intracranial growth of tumors generated from MDA-MB-231/CON and MDA-MB-231/BGN cancer cells, showing different days post-cancer cell implantation as indicated. Due to a strong increase in signal intensity over time, different signal intensity scales were used for displaying images taken on days 6-22, days 27-34, and days 37-41, respectively, to allow for visualization of signals at all time points. [file Image_2.jpeg]
